# Effect of estradiol and cloprostenol combination therapy on expulsion of mummified fetus and subsequent fertility in four crossbred cows

**Published:** 2013

**Authors:** Arumugam Kumaresan, Subhash Chand, Sankaran Suresh, Tushar Kumar Mohanty, Shiv Prasad, Siddhartha Shankar Layek, Kumaresh Behera

**Affiliations:** 1 *Senior Scientist (Animal Reproduction), Cattle Yard, LPM, National Dairy Research Institute, Karnal, India; *; 2 *Technical officer (Veterinary), Cattle Yard, LPM, National Dairy Research Institute, Karnal, India; *; 3 *Veterinary assistant surgeon, Veterinary Dispensary, Tindivanam, India; *; 4 * Principal scientist and in charge, Cattle Yard, LPM, National Dairy Research Institute, Karnal, India; *; 5 * Research Scholar, Cattle Yard, LPM, National Dairy Research Institute, Karnal, India. *

**Keywords:** Cloprostenol, Crossbred cattle, Estradiol, Fertility, Fetal mummification

## Abstract

Four crossbred cows with mummified fetus were utilized for the study. The cows were subjected to gynecological examination and based on the findings the cases were diagnosed as mummified fetus. The cows were treated with 2 mg estradiol valerate and 500 µg cloprostenol and were examined every 12 hr after 24 hr of the treatment for cervical dilatation and other signs related to fetal expulsion. The time duration between treatment and starting of cervical dilatation ranged from 48 to 58h (53.00 ± 2.08 hr). Complete dilatation of cervix was observed after 70.00 ± 2.94 hr post treatment (Range = 64-76 hr). The mean fetal crown-rump length (CRL) was 31.5 cm, which ranged from 27.5 to 38 cm. The number of cotyledons in pregnant horn also showed wide variation (Range 24-38 numbers) with mean ± SE of 30.3 ± 3.07 numbers. In the placenta of three animals irregular shaped large adventitious cotyledons were observed in the inter-cotyledonary areas. Out of the four animals treated, three animals were conceived within three estrous cycles and one animal had cystic ovary in the next cycle and was not conceived even after four cycles. It was concluded that the estradiol and prostaglandin F_2_α (PGF_2_α) combination therapy was effective for expulsion of mummified fetus in crossbred cows without affecting much on future fertility.

## Introduction

Mummification of bovine fetuses is an uncommon condition in cattle in which the fetus dies inside the uterus while the pregnancy is maintained. Generally, the fetus is not expelled and preserved in the uterus beyond the normal gestation period because the pregnancy corpus luteum is maintained beyond the normal gestation period. After death, the fetus usually shrivels up and the amniotic and allantoic fluids are resorbed, dehydrating the fetal tissues and membranes. The immature, unkeratinized skin of the fetus may contribute the mummification process by allowing a faster loss of body.^1^ Two types of mummifications have been encountered in domestic animals, the hematic and the papyraceous type. While the former is seen only in cattle, the papyraceous type occurs in all species.^2^ The incidence of mummification has been reported to be as high as 5% ^3^ but generally less than 2%.^4^ Breed and previous calving history are few factors that predispose fetal mummification.^5^ Higher incidence of mummification has been noticed in Guernsey and Jersey cattle, however, reports on fetal mummification crossbred cattle are very limited. 

Earlier days, estrogen was used to expel the mummified fetus with variable success rate. Roberts, reported an 80% success rate within 3 days with a single injection of estrogen (80 mg of stilbestrol or 5 mg of estradiol 17β).^2^ The use of prostaglandins for treatment of mummification has become routine practice nowadays, however, there are reports that cows with mummified fetus do not always respond to treatment with PGF_2_α.^5^ Though studies are available on the effect of different hormonal treatment on expulsion of mummified fetus, the subsequent fertility of the cattle has not been reported except for very few studies.^5^

The present study reports the effectiveness of low dose estradiol and cloprostenol combination therapy in crossbred cows with mummified fetus and their subsequent fertility. 

## Materials and Methods

**Details about cases. **Four crossbred cows with different stages of fetal mummification (three from smallholder production system and one from organized dairy farm) were utilized for the present study. 

Cow No. 1 (Jersey crossbred) was reared in a village under smallholder production system. The exact date of birth was not available as the farmer did not maintain proper records. The lactation number of the cow was three and fetal mummification occurred in its fourth gestation. As per the routine practice, the cow was fed with locally available grasses and concentrate feed. In most circumstances, the cow was not fed with complete feed as per the requirement. The animal was inseminated with frozen semen of a Jersey bull and diagnosed as pregnant after two months by rectal examination. The cow was referred to a veterinarian with the history of prolonged gestation.

Cows No. 2 and 3 (Holstein Friesian crossbred) were also reared in a village similar to cow No 1. For these cows also the date of birth was not known. The cow No. 2 was in second lactation and fetal mummification was observed in its third gestation. The cow No. 3 was in third lactation and mummification of fetus was observed in her fourth gestation. These cows were artificially inseminated with frozen semen of a Holstein Friesian bull and the animals were brought to the veterinarian for pregnancy check up after 4 and 5 months, respectively. History about the previous calving, based on the farmer’s version, indicated that both these cows had dystocia and the fetus was delivered by traction. Also, both of them had uterine prolapse immediately after calving and were treated by the veterinarian. 

Cow No. 4 (Holstein Friesian X Tharparkar) was a 13 year-old crossbred cow reared in an organized dairy farm (Cattle Yard, National Dairy Research Institute, Karnal, India). The cow was maintained under loose housing and group management system and fed with concentrate and green fodder as per the standard recommendations. During winter, fodders like berseem, oat, mustard and winter maize was fed; whereas in summer and rainy seasons fodders (predominantly maize, sorghum) were given. The history revealed that she had calved eight times and the average milk yield was 4492 kg in 305 days lactation period with a peak yield of 5371 kg. She was a regular breeder and produced a calf per year. However, after the eighth calving she took almost 14 months to conceive. During this period, she showed irregular estrous cycles and was treated with ceftriaxone (Ceft-Plus, Lyka Animal Health Care, Mumbai, India) and antiseptics (Lugol’s iodine). She was brought to the Artificial Insemination Facility of the Institute with the history of prolonged gestation. From the history sheet it was found out that she had already crossed a month period from the expected date of calving. There was no parturition associated changes in the udder as well as pelvis structure. 

**Reproductive examination and treatment regime. **The cows were examined clinically in a routine manner including rectal and vaginal examination and based on the findings the case was diagnosed as fetal mummification. The cows were treated with 2 mg estradiol valerate (Progynon Depot, German Remedies, Maharashtra, India) and 500 µg cloprostenol (Clostenol, Zydus Animal Health Ltd, India) intramuscularly. The cows were examined every 12 hr from 24 hr post-treatment for relaxation of cervix. When the cervix was completely relaxed, the fetus and fetal membranes were removed per vaginum. After unwrapping the fetal membrane from fetus, they were carefully examined for number of cotyledons and presence of adventitious cotyledons.

## Results

Cows No. 1 and 4 had prolonged gestation and the history revealed that both cows had crossed a month period from the expected date of calving (Table 1). Rectal examination revealed that the uterus was located in the pelvic brim and an irregularly shaped, contracted uterus with a fetal mass was palpable. There was no palpable fetal fluid, placentomes and fremitus in the uterine artery. The mass in the uterus was small and very tight in case of cow No. 1. Cows No. 2 and 3 were in the mid of the gestation and were brought for pregnancy diagnosis. Rectal and vaginal examination revealed the similar findings as that of cows No. 1 and 4. Per-vaginal examination of all the cows revealed a closed cervix.

**Fig. 1:**
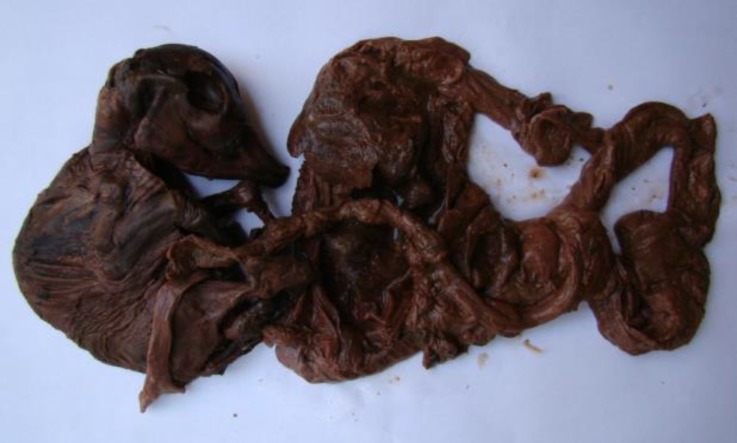
A mummified bovine fetuses immediately after removal

All the four animals responded positively to the estradiol and cloprostenol therapy. The time duration between treatment and starting of cervical dilatation ranged from 48 to 58 hr (53 ± 2.08 hr). Complete dilatation of cervix was observed after 70 ± 2.94 hr after treatment (Range = 64-76 hr). In all the cases the fetus was observed partly in the cervix and partly in the anterior vagina and mild traction was used to remove the fetus along with fetal membranes. The color of the fetus was invariably brown (Fig. 1) and along with the fetus small quantity of viscous chocolate colour fluid was also removed. In all the cases, the fetus was completely wrapped by the fetal membranes. 

The mean fetal crown-rump length was 31.5 cm, which ranged from 27.5 to 38 cm. The number of cotyledons in pregnant horn also showed wide variation (range 24-38 numbers) with mean ± SE of 30.3 ± 3.07 numbers (Table 2). The size of the cotyledons were ranged from 2-5 cm and there were large areas of empty spaces suggestive of absence of cotyledons. In the placenta of three animals irregular shaped large adventitious cotyledons were observed in the inter-cotyledonary areas. Out of the four animals treated, three animals were conceived within three estrous cycles and one animal had cystic ovary in the next cycle and was not conceived even after four cycles.

**Table 1:** Summary of cases of fetal mummification

**No.**	**Breed**	**Age (Years)**	**Number of previous calving**	**History of previous calving**	**Months of pregnancy**
1	Jersey crossbred	7	3	Normal	12
2	Holstein Friesian crossbred	5	2	Dystocia and uterine prolapse	4
3	Holstein Friesian crossbred	7	3	Dystocia and uterine prolapse	5
4	Holstein Friesian X Tharparkar	13	8	Normal	11

**Table 2:** Effectiveness of the treatment and subsequent fertility.

**No.**	**Cervical dilatation** *	**Full relaxation of cervix and fetal removal** *	**Fetal CRL** **(cm) **	**Number of cotyledons in pregnant horn**	**Presence of adventitious placenta**	**Subsequent fertility**
1	54.00	74.00	29.00	38.00	No	Pregnant
2	48.00	64.00	38.00	27.00	Yes	Pregnant
3	58.00	76.00	31.50	24.00	Yes	Pregnant
4	52.00	66.00	27.50	32.00	Yes	Cystic ovary
Mean ± SE 53 ± 2.08		70 ± 2.94	31.5 ± 2.31	30.3 ± 3.07	-	-

* Hours after treatment.

## Discussion

Fetal mummification has been reported in several species, including cattle,^5^ buffaloes,^6^ sheep,^7^ goat,^8^ and pig.^9^ The causes of fetal mummification in cattle are often difficult to identify, however, some genetic factors due to autosomal recessive gene have been reported to be involved in bovine fetal mummification.^2^ Torsion or compression of the umbilical cord, placental defects, infectious agents and abnormal hormonal concentrations are the usual suspect causes behind mummification. Incidence of mummified fetus has been reported in Holstein, Guernsey, Jersey etc.^2^^,^^10^ Several therapeutic regimes for expulsion of mummified fetuses have also been reported, however, information on the subsequent fertility of the affected animal is very much limited.^5^ The present paper discusses the successful treatment of fetal mummification with estradiol and cloprostenol combination in cross bred cattle and their subsequent fertility. 

In the present study, the mummification of fetus was happened from 4 to 4.9 month of pregnancy based on the fetal crown-rump length of fetus. According to Roberts bovine fetal mummification may occur from the 3^rd^ to the 8^th^ months of gestation, and usually is accompanied by slight severe inter-placental hemorrhage, causing a separation of the maternal and fetal placentas.^2^ The fetal membranes wrap around the fetus, and the wall of the uterus firmly encloses fetus, fetal membranes, and a variable amount of gummy, reddish-brown material which stains both the membranes and the fetus. The present study also showed such changes in the fetal placenta. An additional finding observed in the present study was that invariably all the fetal placenta had lower number of cotyledons in the pregnant horn (30.3 ± 3.07) than the values reported innormal pregnancy,^11^ suggesting that it might also be a reason for mummification. It may be hypothesized that though the placental function was tried to compensate by the formation of adventitious placenta, as evidenced by presence of adventitious cotyledons in the fetal placenta in three cases, it might not have been sufficient to fully support the developing fetus and thus led to death and mummification of the fetus. Numerically, cows No. 2 and 3 had lower number of cotyledons in their fetal placenta, which might be attributed to the dystocia and sub-sequent uterine prolapse during last calving as hemorrhage, contamination and damage to uterus during handling of prolapsed uterus may reduce the number of functional caruncles.^2^

All the treated cows responded positively to the treatment and the fetuses were removed manually after 70 ± 2.94 hr of treatment suggesting the effectiveness of estradiol and cloprostenol in expulsion of mummified fetus. Several treatment regimes for expulsion of mummified fetus like use of estradiol,^2^ PGF_2_α and its analogue^10^^,^^12^^,^^13^ and surgical removal^14^^,^^15^ in cattle have been reported with various success rate. The treatment choice of fetal mummification is lysis of corpus luteum (CL) by injection of PGF_2_α, which usually results in satisfactory and safe expulsion of the fetus within 2 to 4 days of treatment.^4^ However, the cows do not always respond to treatment with PGF_2_α.^5^ Hence, it was hypothesized that administration of estradiol would help the cows to respond to PGF_2_α treatment. The results of the present study supported the hypothesis. Estrogens induce lysis of CL by stimulating endogenous PGF_2_α and causes contraction of uterine muscles, relaxation of cervix and expulsion of mummified fetus in cows.^2^ But it has also been reported that the cows treated with estrogen for expulsion of mummified fetus have not been conceived further thus questioning the future fertility of the treated dam.^5^ The study has also mentioned that the dose of estrogen used was exceedingly high (> 4.0 mg), which might have caused the problem. The present study used low dose of estrogen (2 mg of estradiol) to sensitize the endometrium for oxytocin and PGF_2_α as it is well proved that estradiol regulates uterine function by influencing PGF_2_α synthesis via endometrial oxytocin receptors.^16^^,^^17^

The treatment used in the present study was effective in causing cervical dilatation as all the cows had fully dilated cervix at 64-74 hr after treatment, which is in agreement with those reported by Murugavel *et al.* They reported that the cervical dilatation started at 58h after treatment with PGF_2_α analogue and full relaxation of cervix was noted at 72 hr after treatment in cow with mummified fetus.^13^ Even though the birth canal was relaxed sufficient to allow the expulsion of fetus, invariably in all the cases the fetus was observed partly in the cervix and partly in anterior vagina and removed manually with mild traction, suggesting a uterine contractility defect. Due to the long time atony of the uterus and dry, firm and leathery fetus, the mummified fetus may not be expelled by the dam on its own and thus application of gradual traction is recommended for removal of mummified fetus.^2^ Among the four cows treated for mummified fetus, three of them conceived in three estruses after treatment, while one cow had cystic ovary and not conceived even after four cycles. These findings are in contrary to those reported by Lefebvre *et al*.^5^ They reported no conception in cows treated with PGF_2_α and estrogen or oxytocin for expulsion of mummified fetus and the possible reason attributed was high dose of estrogen. The present study used low dose of estrogen, and found effective when administered along with PGF_2_α for expulsion of mummified fetus and also it did not affect conception as three of the treated animals were conceived. One treated cow had cystic ovary, which might have been due to the individual’s threshold for estrogen or some other reasons.

It may be concluded, from the findings of the study, that the number of cotyledons in the pregnant horn of the mummified fetus is lower than the normal and low dose estradiol and PGF_2_α combination therapy is effective for expulsion of mummified fetus in crossbred cows without compromising much on future fertility.
